# Southward spreading of the Fukushima-derived radiocesium across the Kuroshio Extension in the North Pacific

**DOI:** 10.1038/srep04276

**Published:** 2014-03-04

**Authors:** Yuichiro Kumamoto, Michio Aoyama, Yasunori Hamajima, Tatsuo Aono, Shinya Kouketsu, Akihiko Murata, Takeshi Kawano

**Affiliations:** 1 Research Institute for Global Change, Japan Agency for Marine-Earth Science and Technology, 2-15 Natushima-cho, Yokosuka, Kanagawa 237-0061, Japan; 2 Geochemical Research Department, Meteorological Research Institute, 1-1 Nagamine, Tsukuba, Ibaraki 305-0052, Japan; 3 Low Level Radioactivity Laboratory, Kanazawa University, Wake, Nomi, Ishikawa 923-1224, Japan; 4 National Institute of Radiological Sciences, 4-9-1 Anagawa, Inage-ku, Chiba, Chiba 263-8555, Japan; 5 Current address: Institute of Environmental Radioactivity, Fukushima University, 1-1 Kanayagawa, Fukushima, Fukushima 960-1296, Japan.

## Abstract

The accident of the Fukushima Dai-ichi nuclear power plant in March 2011 released a large amount of radiocesium into the North Pacific Ocean. Vertical distributions of Fukushima-derived radiocesium were measured at stations along the 149°E meridian in the western North Pacific during the winter of 2012. In the subtropical region, to the south of the Kuroshio Extension, we found a subsurface radiocesium maximum at a depth of about 300 m. It is concluded that atmospheric-deposited radiocesium south of the Kuroshio Extension just after the accident had been transported not only eastward along with surface currents but also southward due to formation/subduction of subtropical mode waters within about 10 months after the accident. The total amount of decay-corrected ^134^Cs in the mode water was an estimated about 6 PBq corresponding to 10–60% of the total inventory of Fukushima-derived ^134^Cs in the North Pacific Ocean.

The massive Tohoku earthquake and consequent giant tsunamis on 11 March 2011 resulted in serious damage to the Fukushima Dai-ichi nuclear power plant (FNPP1)^1^. Radiocesium (^134^Cs and ^137^Cs) derived from the damaged FNPP1 caused radioactive contamination of the islands of Japan and the North Pacific Ocean^2^. Most of the Fukushima-derived radiocesium deposited on land has remained in soils. Within about 100 km of the FNPP1, where contamination was serious, the radiocesium in soils has been measured intensively^3^. The decay-corrected ratio of ^134^Cs/^137^Cs in soils has been calculated to be 1.0, which suggests that the total amounts of ^134^Cs and ^137^Cs released from FNPP1 were equivalent. The relationship between the radiocesium activity in the soil and the air dose rate derived from airborne monitoring has provided a map of the density of radiocesium deposition throughout the islands of Japan^4^. The sum of the deposition, the total inventory of ^137^Cs (or ^134^Cs) on the islands of Japan, has been estimated to be 2.4 PBq^5^. However, the total amount of Fukushima-derived radiocesium in the North Pacific remains uncertain, because it has been difficult to obtain sufficient samples of water, especially from subsurface and deep waters, in the vast North Pacific Ocean, except from the coastal area near the FNPP1^6^,^7^,^8^.

Radiocesium isotopes were released into the North Pacific through two major pathways, direct discharges of radioactive water and atmospheric deposition. About ten days after the earthquake, Tokyo Electric Power Company and the Ministry of Education, Culture, Sports, Science and Technology of Japan (MEXT) began marine monitoring in the coastal area within about 50 km from the FNPP1^6^,^7^,^8^. These high-frequency measurements have facilitated an evaluation of the total amount of radiocesium derived from the directly discharged radioactive water. The values estimated in several studies were in the range 4–6 PBq^1^,^9^,^10^,^11^,^12^,^13^, although one study calculated the value to be 27 PBq (12–41 PBq)^14^. The total direct release of 27 PBq was somewhat of an overestimate^11^,^15^ and resulted in activities in a model ocean that were unrealistically high compared to activities measured in the real ocean^16^. However, radiocesium activities measured during a cruise in June 2011, mainly in the open ocean^17^, indicated that the total activity of ^137^Cs (or ^134^Cs) directly discharged to the ocean equaled 11–16 PBq^18^,^19^.

A large portion of the radiocesium released to the atmosphere from the FNPP1 was deposited onto the North Pacific Ocean, because the winds over Japan usually blow from the west in the spring^20^. However, the small number of observational data in the open ocean cannot estimate the total oceanic deposition directly. Alternatively, that could be calculated indirectly from the total amount of radiocesium released to the atmosphere, which was derived primarily from measurements on land. Estimations of the total amount released to the atmosphere range widely, from 8.8 to 37 PBq^1^,^5^,^9^,^11^,^14^,^21^,^22^,^23^,^24^,^25^. The 2.4 PBq deposited onto the islands of Japan suggests that most of the remaining radiocesium, 6.4–35 PBq, found its way into the North Pacific through atmospheric deposition. Atmospheric models have estimated independently the total oceanic deposition to be 5.8–30 PBq^5^,^9^,^11^,^12^,^23^,^25^, similar to the range of 6.4–35 PBq. However, the deposition on land has been overestimated in many of the models.

Efforts to obtain observational data from the open ocean have continued. The marine monitoring from March 2011 by MEXT or the Nuclear Regulation Authority was extended eastward to the 144°E meridian in August 2011^7^. Radiocesium measurements in the area further east have been reported in several publications^8^,^17^,^26^,^27^,^28^,^29^,^30^,^31^. Seawater sampling from April 2011 during commercial ship cruises has produced a valuable dataset across the North Pacific^28^, although as in many other previous studies, most of the samples were collected only at the surface. In June 2011 vertical profiles of the Fukushima-derived radiocesium were measured at stations along 147°E between 34.5°N and 38°N, and it was found that the radiocesium had penetrated to a depth of about 200 m roughly two months after the disaster^17^. Although these observational data are still insufficient for direct estimation of the total amount of radiocesium in the whole North Pacific, these data can be used to validate ocean model simulations that have predicted vertical and horizontal spreading of the radiocesium in the ocean^13^,^15^,^16^,^25^,^32^,^33^.

Here we report the vertical distributions of the Fukushima-derived radiocesium at stations along 149°E between 10°N and 42°N in the winter of 2012, about ten months after the accident. Our preliminary reports, which have already been published^31^,^34^, revealed that (1) the Fukushima-derived radiocesium activity was highest in the transition area between the subarctic and subtropical regions and (2) the radiocesium was transported southward across the Kuroshio Extension (KE) through subsurface layers. In this study, we discuss the causes of the southward spreading of the radiocesium based on temporal changes in the activity of surface waters. Secondly, we have estimated the vertical water-column inventory of radiocesium. These results will contribute to determination of the total inventory of radiocesium and will facilitate prediction of the spreading of the Fukushima-derived radiocesium in the North Pacific Ocean in the future. We measured both ^134^Cs and ^137^Cs activities (Methods). The ratio of decay-corrected ^134^Cs/^137^Cs in samples in which the ^137^Cs activity was higher than 20 Bq m^−3^ was about 0.95. The small excess of ^137^Cs was derived from another source of ^137^Cs, global fallout due to the nuclear bomb testing in the 1950s and 1960s^35^. The excess ^137^Cs in surface waters (about 1.5 Bq m^−3^) in the winter of 2012 corresponds to bomb-produced ^137^Cs activities (about 1.9 Bq m^−3^) in surface water of the North Pacific before the accident (about 2.4 Bq m^−3^ in 2000)^36^. Therefore, only results for ^134^Cs, which is a unique tracer of the FNPP1 accident, are presented in later sections.

## Results

### Temporal changes in ^134^Cs activity in surface waters

**Figure 1:**
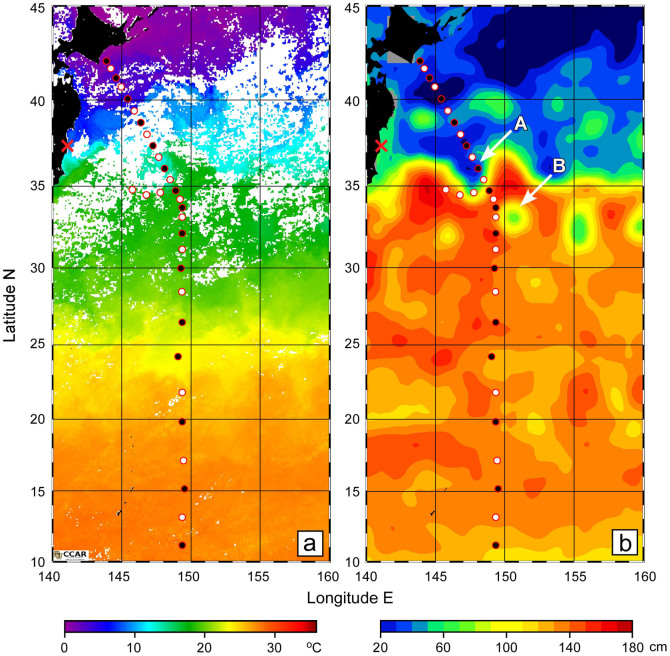
Water sampling locations for radiocesium measurements superimposed on backgrounds of (a) sea surface temperature (SST, °C) and (b) sea surface height (SSH, cm). White and black circles denote stations for surface sampling only and a deep hydrocast to a depth of 800 m, respectively. The red cross shows the location of the Fukushima Dai-ichi nuclear power plant. The SST was derived from Moderate Resolution Imaging Spectroradiometer data averaged between 15 January 2012 and 14 February 2012 (Level-3, Terra, 4-km resolution). The images of SST were produced by the Colorado Center for Astrodynamics Research Data Viewer. The SSH map is based on one-week average gridded data (1/3° × 1/3°) for 1 February 2012; they were produced by the Segment Sol Multimissions d'Altimétrie d'Orbitographie et de Localisation Précise/Data Unification and Altimeter Combination System and distributed by the Archiving, Validation and Interpretation of Satellites Oceanographic Data with support from the Centre National d'Etudes Spatiales. The maps in this figure were drawn using Ocean Data View^54^.

Our sampling stations were located in the western North Pacific from cold subarctic to warm tropical regions, although information on sea surface temperatures estimated by satellite sensors was patchy in the northern area due to cloudy conditions during the sampling cruise (Figure 1a). The image of sea surface height (SSH) implied that our observational line along 149°E crossed eastward-flowing currents around 35°N and 40°N where SSH gradient was relatively steep (Figure 1b). The northern and southern currents correspond to the subarctic and KE fronts, respectively. Here we define areas north of the subarctic and south of the KE fronts as the subarctic and subtropical regions, respectively. In addition, we designate the area between the two fronts as the transition area, in which the FNPP1 is situated (Figure 1). Although a boundary between the subtropical and tropical regions is not clear in Figure 1, we provisionally regarded the area south of 20°N as the tropical region because of the subtropical front around 20°N^37^. The distribution of SSH also suggests that the observational line crossed a southward meander of the KE front around 148°E (A in Figure 1b).

**Figure 2:**
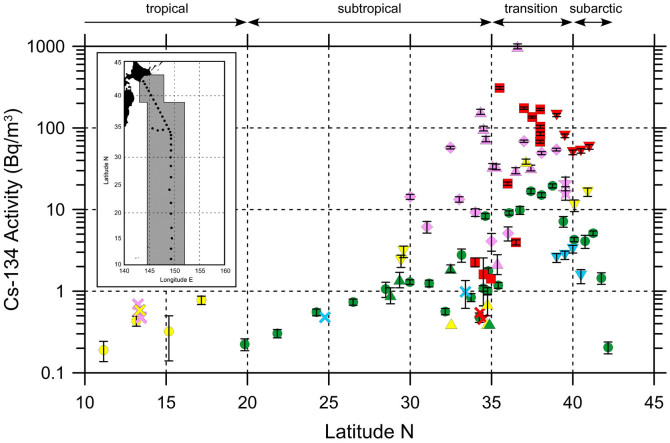
^134^Cs activity (Bq m^−3^) in surface seawaters of the western North Pacific from April 2011 to September 2012. The activity was corrected to the date of sampling. Pink, red, yellow, green, and blue symbols denote the activities in April–May 2011, June–August 2011, September–December 2011, January–March 2012, and April–September 2012, respectively. The data are from Honda *et al.* (2012)^26^ (diamonds), Buesseler *et al.* (2012)^17^(squares), Karasev (2012)^27^ (stars), Aoyama *et al.* (2013)^28^ (triangles), Kaeriyama *et al.* (2013)^29^ (inverted-triangles), Kamenik *et al.* (2013)^30^ (crosses), and this work (circles). Symbols without an error bar show the detection limits of analyses; their ^134^Cs activities were less than the detection limit. Dots and the shaded area on the map show the sampling locations of this work in the winter of 2012 and the area between approximately 145°E and 152°E sampled during previous studies, respectively. The map in this figure were drawn using Ocean Data View^54^.

In surface seawaters, Fukushima-derived ^134^Cs activity was detected at all the stations along the 149°E meridian from the subarctic to tropical regions in the winter of 2012 (Figure 2). The radioactivity was highest (10–20 Bq m^−3^) in the transition area between 35°N and 40°N. In the subarctic region, north of 40°N, the activity decreased sharply at higher latitudes and fell to about 0.2 Bq m^−3^ at the northernmost station. To the south of the KE, between approximately 30°N and 35°N, the activity declined to a few Bq m^−3^ and then dropped to less than 1 Bq m^−3^ farther south of 30°N. We also collected seawater samples along a zonal transect at approximately 35°N, which crossed the southward meander of the KE (A in Figure 1b). Relatively high activity (about 8 Bq m^−3^) was observed at a station at 148°E, near the approximate center of the meander.

To discuss temporal changes in the surface ^134^Cs activity, we also show in Figure 2 the activities measured in surface waters (0–20 m depth) between approximately 145°E and 152°E during previous studies^17^,^26^,^27^,^28^,^29^,^30^. Just after the accident, in April–May 2011, the activities between 30°N and 40°N were high, though the range of activity was large (approximately 2–1000 Bq m^−3^). In the transition area (35°N–40°N), the activity increased significantly in the following period, June–August 2011. After that time, the activity decreased piecemeal and then fell to a few Bq m^−3^ in August 2012. The surface activity in the subarctic region to the north of 40°N also decreased monotonically from about 50 to a few Bq m^−3^ between June 2011 and August 2012. The transitory increase during June–August 2011, which was observed in the transition area, was indistinct in the subarctic region because of a lack of data in April–May 2011. To the south of the KE, between 30°N and 35°N, the high surface activity in April–May 2011 quickly decreased to a few Bq m^−3^ by June 2011. The magnitude of the temporal change of activity in the surface waters to the south of 30°N, including the southern subtropical and tropical regions, is uncertain, because ^134^Cs activity was detected only in the winter of 2012. ^134^Cs has a short half-life of only 2.07 years, and the activity decay-corrected to the sampling date decreased by 50–75% from April 2011 to September 2012. The fact that the observed activity decreased at a rate faster than the radioactive decay rate suggests that the surface ^134^Cs activity was diluted by advection and diffusion.

### Vertical profiles and inventories of ^134^Cs activity

**Figure 3:**
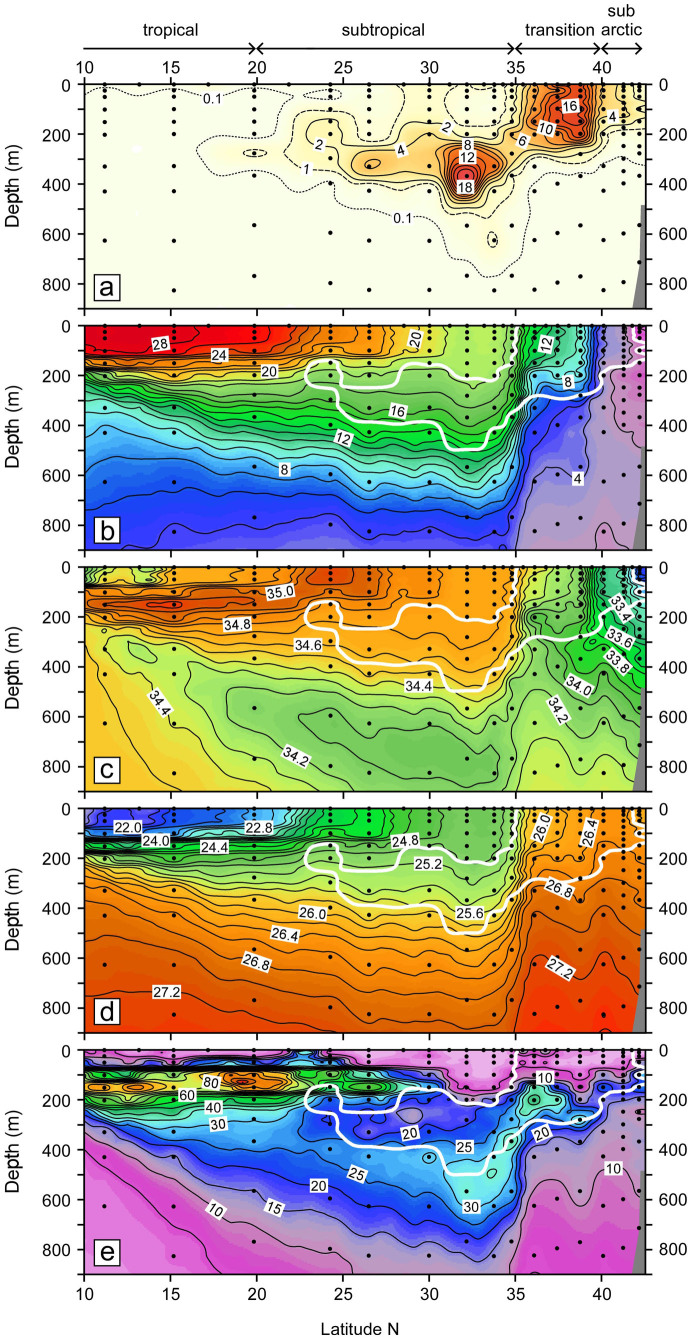
Cross sectional views of ^134^Cs activity (a, Bq m^−3^), potential temperature (b, °C), practical salinity (c), potential density anomaly or σ_θ_ (d, kg m^−3^), and potential vorticity (e, 10^−11^ m^−1^ s^−1^) along approximately 149°E in the winter of 2012. Contour intervals in (a), (b), (c), (d), and (e) are 2 Bq m^−3^, 1°C, 0.1, 0.2 kg m^−3^, and 5 × 10^−11^ m^−1^ s^−1^, respectively, except for broken (1 Bq m^−3^) and dotted (0.1 Bq m^−3^) lines in (a). Dots show points sampled for radiocesium activity measurements. Thick white lines in (b), (c), (d), and (e) indicate isolines of 2 Bq m^−3^ of ^134^Cs activity. All data in this figure, except potential vorticity, are listed in Supplementary Table 1 together with the ^137^Cs data. This figure was drawn using Ocean Data View^54^.

In the transition area between 35°N and 40°N, where surface ^134^Cs activity was highest, ^134^Cs activity from the surface to a depth of about 200 m was almost constant (Figure 3a). The homogeneity of the activity in the surface layer reflects surface cooling and vertical mixing in the winter and is consistent with the vertical uniformity of water temperature, salinity, density, and therefore the small potential vorticity at that time (Figs. 3b–3e). The activity then decreased sharply just below the winter mixed layer. The ^134^Cs had penetrated to a depth of about 300 m by the winter of 2012. In the subarctic region, the ^134^Cs activity in the surface mixed layer was also almost uniform vertically but lower than in the transition area. The depth of penetration was shallower than in the transition area, probably because the mixed layer was shallower, about 150 m deep. At the northernmost station, the activity in the mixed layer were lower as in the surface water. The vertical profiles of ^134^Cs activity in the transition area and subarctic region can be largely explained by vertical diffusion between the surface mixed layer and deeper layers.

To the south of the KE, the surface activity was less than a few Bq m^−3^ in the winter of 2012 (Figure 2). Figure 3a indicates that the ^134^Cs activity was also low (but significantly above the detection limit) in the surface mixed layer from the surface to a depth of 150–200 m between approximately 25°N and 35°N. In contrast, to the south of 20°N the activity was not detected in the surface mixed layer to a depth of 100–150 m, except in surface waters collected with a bucket. Below the surface mixed layer, we found a conspicuous subsurface maximum centered at a depth of about 300 m throughout the subtropical region between 20°N and 35°N. This subsurface tongue-shaped maximum appeared in a pycnostad between potential density anomalies of approximately 25.0 and 25.6 σ_θ _(Figure 3d), which corresponds to water temperatures of 15–18 °C (Figure 3b) and salinities of approximately 34.60–34.75 (Figure 3c). The pycnostad resulted in a subsurface minimum of potential vorticity (Figure 3e). Higher activities in the subsurface maximum were observed at 32°N and 34°N (10–20 Bq m^−3^), and the activity decreased at lower latitudes. We also note that the ^134^Cs had penetrated into deeper layers, to depths of at least 600 m, between 32°N and 35°N.

**Figure 4:**
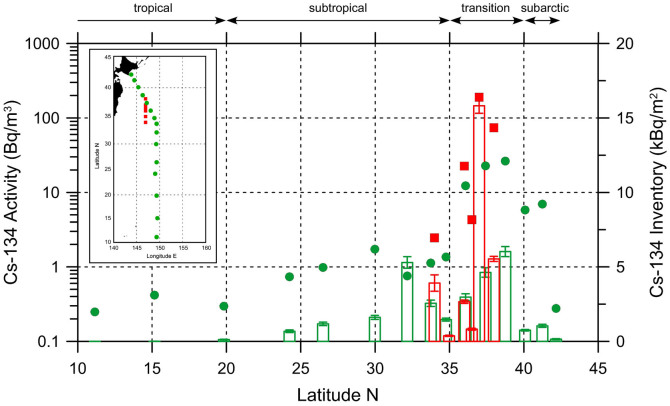
Vertically integrated (areal) inventories of ^134^Cs (kBq m^−2^, right ordinate) in the western North Pacific. Green and red histograms indicate inventories at the 15 stations along approximately 149°E in the winter of 2012 and at 6 stations along 147°E from 34°N to 38°N in June 2011^17^, respectively. Error bars on the tops of histograms indicate uncertainties (standard deviations). The ^134^Cs activities (Bq m^−3^, left ordinate) in surface seawater in the winter of 2012 (green circles) and June 2011^17^ (red squares) are also shown. The activities and inventories have been corrected to 11 March 2011. The map in this figure were drawn using Ocean Data View^54^.

We calculated vertically integrated (i.e., areal) ^134^Cs inventories from the surface to a depth of 800 m in the winter of 2012 (Figure 4). The areal inventories were corrected for radioactive decay to the date of the earthquake, 11 March 2011. High areal inventories were observed in the transition area, where surface activities were also high. Although the surface activities were low in the subtropical region between 30°N and 35°N, the areal inventories were comparable to those in the transition area because of the subsurface activity maximum. The areal inventories of ^134^Cs activity in the subarctic region (40°N–42°N), transition area (35°N–40°N), and subtropical region (20°N–35°N) were calculated to be 0.8 ± 0.1, 4.6 ± 0.3, and 1.6 ± 0.1 kBq m^−2^, respectively, where the error bounds indicate standard deviations. We compared the areal inventories in the winter of 2012 with those calculated about 8 month earlier, in June 2011^17^ (Figure 4). The areal inventory in the transition area (36°N–38°N) in June 2011, 7.9 ± 0.3 kBq m^−2^, implies about a 40% decrease in the areal inventory between June 2011 and the winter of 2012, although the spatial variation in June 2011 was larger than in the winter of 2012. The mean of the decay-corrected radioactivity in the surface water also decreased by about 70%, from 73 to 21 Bq m^−3^, in the transition area during the same period. The higher rate of decline in the surface ^134^Cs radioactivity was caused by its deeper penetration during the winter of 2012 (to a depth of about 300 m) than in June 2011 (to a depth of about 200 m). A relatively large areal inventory at the southernmost station (36°N) to the south of the KE in June 2011 was caused by a subsurface ^134^Cs maximum at depths of 150–450 m.

## Discussion

In April–May 2011, just after the accident, the ^134^Cs activity was as high as 1000 Bq m^−3^ in the surface waters of the transition area and just to the south of the KE (30°N–40°N) along approximately 145°E–152°E, more than 500 km from the FNPP1 (Figure 2). In April 2011, ^134^Cs activity was also observed at stations in the subarctic and subtropical regions, more than 1000 km distant from the plant^26^,^28^. The wide dispersal of Fukushima-derived ^134^Cs in the western North Pacific within about two months of the accident is consistent with patterns of atmospheric deposition of ^134^Cs simulated by atmospheric models^13^,^25^,^38^. A low-pressure system traveling across Japan from 14–15 March 2011 was found to be effective in lifting particles containing ^134^Cs from the surface layer to the altitude of the westerly jet stream, which carried the particles across the North Pacific within 3–4 days^39^.

**Figure 5:**
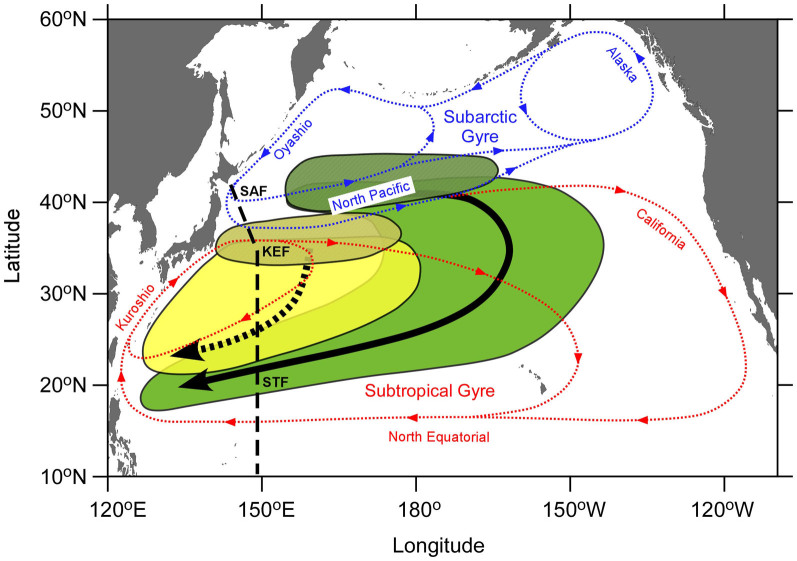
A schematic view of formation and subduction of mode waters in the North Pacific. Yellow and yellow-shaded ellipses indicate spreading and formation areas, respectively, of STMW (25.0–25.6 σ_θ_). Green and green-shaded areas indicate spreading and formation areas, respectively, of CMW (26.0–26.6 σ_θ_), which is denser than STMW. Thick broken and solid arrows show spreading directions of STMW and CMW, respectively. Blue and red dotted lines are surface water currents of the subarctic and subtropical gyres, respectively. The broken line denotes our observational line at 149°E in the winter of 2012. SAF, KEF, and STF indicate the subarctic, Kuroshio Extension, and subtropical fronts along the observational line, respectively. The map in this figure were drawn using Ocean Data View^54^ and this figure has been modified from one in the literature^55^.

In the transition area between 35°N and 40°N, the ^134^Cs activities in surface waters during June–August 2011 were significantly higher than in April–May 2011 (Figure 2), which implied that contaminated waters discharged from the FNPP1 had been transported by the eastward-flowing North Pacific Current (Figure 5). The radiocesium activities in surface seawater collected by commercial cruise ships revealed an eastward propagation of the main plume of the directly discharged ^134^Cs. The zonal speed of the plume was estimated to be about 200 km month^−1^, a speed that was consistent with trajectories of Argo floats launched near the FNPP1^28^. Therefore, arrival of the directly discharged ^134^Cs water in June–August 2011 was delayed by about two months relative to the atmospheric deposition in April–May 2011. The activity decrease in September–December 2011 indicated that the main body of the plume had passed to the east between April–May and September–December 2011. The radiocesium, however, also had spread vertically and penetrated deeper in the winter of 2012 (a depth of about 300 m) compared to June 2011 (a depth of about 200 m).

The ^134^Cs activity in the subarctic region was lower than in the transition area throughout the observational period; its pattern of temporal change, however, was similar to that in the transition area (Figure 2). Whether there were intrusions of directly discharged ^134^Cs from the transition area to the subarctic region is unclear, because the transitory increase in June–August 2011 was obscure in the subarctic region. Off the Kuril Islands, the activities in the surface waters of the Oyashio Current, which flows into the subarctic region (Figure 5), were less than a few Bq m^−3^ in April 2011^27^. If the supply of directly discharged ^134^Cs to the subarctic region had been blocked by the subarctic front, the surface activity in the subarctic region would have dropped more sharply because of the inflow of Oyashio Current water, the ^134^Cs activity of which was low. In fact, the low activity at the northernmost station in the winter of 2012 implies an intrusion of Oyashio Current water (Figure 3a). Therefore, it is likely that the directly discharged ^134^Cs was transported into the subarctic region through water exchanges between the transition area and the subarctic region. The gradual decrease of surface ^134^Cs in the subarctic region indicates that the directly discharged ^134^Cs was transported eastward and diffused vertically over time, as was also the case in the transition area.

Between 30°N and 35°N in the subtropical region, the ^134^Cs derived from atmospheric deposition during April–May 2011 was apparently swept out in June–August 2011 (Figure 2). In May 2011, Fukushima-derived ^134^Cs was not detected in surface waters just south of Japan^28^, where the Kuroshio Current (the upper stream of the KE) flows northeastward (Figure 5). This low ^134^Cs activity in the Kuroshio Current region suggests that a new and relatively “clean” KE current from the west probably flushed out the ^134^Cs in the surface water between 30°N and 35°N. This process was also clearly demonstrated in ocean model simulations^12^,^13^ and suggests that an exchange of surface seawater between the transition area and the subtropical region was restrained by the KE front. The ^134^Cs activity in the surface mixed layer between 25°N and 35°N was low but detectable in the winter of 2012 (Figure 3a). The ^134^Cs derived from atmospheric deposition just after the accident probably recirculated within the western subtropical region (Figure 5). Alternatively, the ^134^Cs in the mixed layer could be explained by entrainment of ^134^Cs from the subsurface maximum just below the mixed layer. To the south of 20°N, the ^134^Cs was detected only in surface waters collected with a bucket. Although the cause of those surface activities is not sure, a little contamination on the bucket is possible.

In the subtropical region between 20°N and 35°N, we found a subsurface ^134^Cs maximum just below the surface mixed layer in the winter of 2012 (Figure 3a). This tongue-shaped subsurface plume appeared on a pycnostad between 25.0 and 25.6 σ_θ_ (Figure 3d) that resulted in a subsurface minimum of potential vorticity in the corresponding layers (Figure 3e). We conclude that the ^134^Cs subsurface maximum was derived from formation and subduction of Subtropical Mode Water (STMW)^40^. To the south of the KE between approximately 30°N and 35°N, STMW is formed and penetrates to a depth of about 400 m (25.6 σ_θ_) in late winter. This STMW then spreads to nearly the subtropical front^35^ through advection over the Kuroshio recirculation region^41^,^42^ (Figure 5). Atmospheric deposition of the Fukushima-derived ^134^Cs in the North Pacific Ocean occurred mainly in March 2011, when STMW was just being formed. Therefore, the ^134^Cs deposited just to the south of the KE was probably mixed vertically to depths of 300–400 m immediately. The high activities in the ^134^Cs subsurface plume at 32°N and 34°N (10–20 Bq m^−3^) were nearly identical with those in the surface waters between 30°N and 35°N in April–May 2011 (Figure 2). One could argue that the high subsurface activities in the winter of 2012 were remnants of the ^134^Cs that penetrated deeply during March 2011. The ^134^Cs in newly formed STMW then started to spread to around 20°N along subsurface isopycnals (25.0–25.6 σ_θ_). In June–August 2011, the ^134^Cs in the surface mixed layer between 30°N and 35°N may have been flushed out and the subsurface plume appeared between 20°N and 35°N (Figure 3a). The subsurface maximum observed at 36°N to the south of the KE in June 2011^17^ is consistent with the immediate subduction of the Fukushima-derived ^134^Cs.

The deeper penetration of ^134^Cs to depths of about 600 m (26.6 σ_θ_) between 32°N and 35°N (Figure 3a) cannot be explained by formation of STMW, the deepest convection of which is to about 400 m (25.6 σ_θ_). The penetration of the ^134^Cs to 26.0–26.6 σ_θ_ is reminiscent of ventilation of another, denser mode water in the North Pacific, the Central Mode Water (CMW)^43^. The formation area of CMW is situated in the transition area in the central North Pacific. The CMW spreads eastward along the North Pacific Current, turns southward, and then turns westward (Figure 5). Despite its similar water density anomaly (26.0–26.6 σ_θ_), the path of the CMW as it spreads is likely to be to the south of approximately 30°N, along 149°E. In addition, a transit time as short as about 10 months (between March 2011 and January 2012) from the formation area to 149°E longitude is not plausible, because the renewal time of CMW is more than 20 years^44^.

Another possible explanation for the deeper penetration is conveyance of ^134^Cs from the transition area across the KE. The satellite image of SSH indicates that stations at 32°N and 34°N were located near a cyclonic eddy centered at 33°N, 151°E (B in Figure 1b). This cyclonic eddy originated in a southward meander of the KE front around 158°E and pinched off southward from the meander in September 2011. Then the eddy moved westward and reached 151°E in January 2012. Similar to the relatively high activity at the station located near the center of the southward meander of the KE at 148°E (A in Figure 1b), the cyclonic eddy probably consisted of denser waters with a higher activity of ^134^Cs, because the surface ^134^Cs activity in the source area (the transition area) was more than 50 Bq m^−3^ in October 2011^29^. A model simulation has indicated that a cyclonic eddy detached from the KE front holds the transition area water in it, while small leakage occurs from layers denser than 26.0 σ_θ_^45^. Although the vertical profiles of temperature and salinity do not indicate the presence of a cyclonic eddy between 32°N and 34°N (Figs. 3b and 3c), a small amount of leakage of ^134^Cs from such an eddy could explain the deeper penetration of the ^134^Cs (Figure 3a). Alternatively, the deeper penetration can be attributed to direct advection along subsurface isopycnals from the transition area. A salinity minimum observed just south of the KE has been explained by intrusion of Oyashio low-salinity water in the transition area; this intrusion was associated with the frontal wave structure of the KE^46^,^47^. The deeper ^134^Cs penetration just south of the KE (Figure 3a) implies that a similar subsurface intrusion occurred in the winter of 2012.

In the winter of 2012 the areal inventory of ^134^Cs (decay-corrected to the date of the accident) in the subtropical region (20°N–35°N) was estimated to be 1.6 ± 0.1 kBq m^−2^, which is about one-third of the areal inventory in the transition area (35°N–40°N), 4.6 ± 0.3 kBq m^−2^ (Figure 4). The integral of the areal inventory along the meridian in the subtropical region, however, was 2.7 ± 0.1 GBq m^−1^, which was about twice the value of the integral in the transition area, 1.4 ± 0.1 GBq m^−1^. The large inventory in the subtropical region suggests that the ^134^Cs released from the FNPP1 had been transported not only eastward but also southward. The average activity of the decay-corrected ^134^Cs in the STMW was 5.6 ± 0.4 Bq m^−3^. We here assumed that this average activity could be regarded as the mean activity of the whole STMW in the North Pacific, because our observational line was located near the center of the area of STMW (Figure 5). An estimation of the total volume of STMW (about 1 × 10^6^ km^3^)^44^ implies that the STMW contained about 6 PBq of ^134^Cs. Estimates of the total ^134^Cs released to the North Pacific Ocean ranged from 10 PBq (direct discharge of 4 PBq + atmospheric deposition 6 PBq) to 46 PBq (16 + 30 PBq). Thus, the 6 PBq inventory accounts for 10–60% of the total release. However, the total inventory in the subtropical region derived from the activity in STMW may be underestimated, because CMW probably carried the radiocesium into the subtropical region, too (Figure 5).

In this study we reconstructed the temporal change in Fukushima-derived radiocesium in surface water of the western North Pacific during about one year and a half after the accident. In April–May 2011 the ^134^Cs activity between 30°N and 40°N arose from the atmospheric deposition (Figure 2). In the north of the KE front, the transition area and subarctic region the discharged ^134^Cs was added while in the south of the KE front the atmospheric-deposited ^134^Cs was flushed out by the KE current during the following period. We found the subsurface maximum of ^134^Cs in the subtropical region about 10 months after the accident. The radiocesium that entered the ocean just south of the KE front via atmospheric deposition was subducted southward immediately because of formation of STMW. This process is reminiscent of the southward spreading of radiocesium derived from the nuclear bomb testing in the North Pacific via STMW formation^48^. In addition, there is an indication that the Fukushima-derived radiocesium in the transition area was conveyed southward across the KE by cyclonic eddies that detached from the KE and by subsurface intrusion under the KE. The rapid southward spreading of the ^134^Cs through subsurface layers seems to not have been simulated well in ocean models^13^,^15^,^16^,^32^,^33^, probably because of problems associated with the simulation of processes responsible for formation/subduction of STMW in these models. The estimated inventory in the subtropical region (6 PBq or 10–60% of the total inventory) is probably a lower limit of estimation because contribution of CMW was not counted. The results in this study clearly suggest that radiocesium released from FNPP1 into the North Pacific Ocean had been transported not only eastward along with the surface currents but also southward due to formation/subduction of STMW within about 10 months after the accident.

## Methods

### Seawater sampling

Seawater samples for radiocesium measurements were collected during a cruise of the Research Vessel MIRAI (MR11-08) from December 2011 to February 2012. This cruise also served as a repeat hydrography along one of observation lines of the World Ocean Circulation Experiment (WOCE) in the western Pacific Ocean, specifically the WOCE-P10/P10N line, which follows the 149°E meridian approximately. We collected seawater at 31 stations along the line between 10°N and 42°N (Figure 1). Surface samples were taken from the deck with a bucket or by pumping water from directly beneath the ship (a depth of about 4 m). The temperature and salinity of the surface water in the bucket were measured with a calibrated mercury thermometer and a salinometer (Autosal model 8400, Guildline Instruments), respectively. The temperature and salinity of the pumped water were measured with a sensor system for conductivity (or salinity), temperature, and pressure (SBE-11plus, Sea-Bird Electronics, Inc.). The salinity sensor on the system was calibrated with bottled seawater, the salinity of which had been measured with the salinometer. At 15 of the 31 stations, deeper seawater from depths of 25 to 800 m was collected with 12-liter, polyvinyl chloride bottles (Model 1010X NISKIN-X, General Oceanics, Inc.) equipped with another sensor system (SBE-11plus, Sea-Bird Electronics, Inc.). We collected about 20 dm^3^ of seawater from each depth. The seawater was filtered through a 0.45 μm pore size membrane filter (HAWP14250, Millipore) and acidified on board by adding 40 cm^3^ of concentrated nitric acid (Nitric Acid 70% AR, RCI Labscan, Ltd.) within 24 h after sampling.

### Sample preparation

After the cruise, radiocesium in the seawater sample was concentrated on ammonium phosphomolybdate (AMP) in onshore laboratories for measurement of gamma-ray activity. The sample preparation was conducted in laboratories of four agencies: the Japan Agency for Marine-Earth Science and Technology (JAMSTEC), the General Environmental Technos Co., Ltd. (KANSO), the Japan Marine Science Foundation (JMSF), and the National Institute of Radiological Sciences (NIRS). In the former two laboratories, the pH of the seawater sample was adjusted to 1.6, and 0.26 (or 0.39) g of cesium chloride (>98.0%, KANTO Chemical Co., Inc.) was added to the seawater as a carrier. Then 4 (or 6) g of AMP, made from hexaammonium heptamolybdate tetrahydrate (>98.0%, KANTO Chemical Co., Inc.) and phosphoric acid (85%, Wako Pure Chemical Industries, Ltd.), was added to the seawater and mixed well for two hours to form an AMP/Cs compound. The compound was stored overnight and then filtered onto a paper filter (Quantitative Filters Papers 5C, Tokyo Roshi Kaisha, Ltd.). After drying at room temperature, the compound on the filter was transferred to a teflon tube (5 cm^3^) for gamma-ray measurement. The recovery of radiocesium from the seawater into the AMP/Cs compound in the tube was estimated to be about 95%. These procedures basically follow a protocol described in the literature^49^. The JMSF and NIRS laboratories used similar AMP methods^50^,^51^. The recoveries of radiocesium at the JMSF and NIRS laboratories were about 95 and 91%, respectively.

### Analyses

The radiocesium activity in the AMP/Cs compound was measured in the laboratories of the Mutsu Oceanographic Institute/JAMSTEC, Low Level Radioactivity Laboratory/Kanazawa University (LLRL/KU), and the NIRS. In JAMSTEC, the radiocesium was measured with low-background Ge-detectors (Well-type GCW2022-7915-30-ULB, Canberra Industries, Inc.), which were calibrated with gamma-ray volume sources (Eckert & Ziegler Isotope Products) certificated by Deutscher Kalibrierdienst (DKD). The gamma counting time ranged from a day to a week, and ^134^Cs and ^137^Cs activities were evaluated from gamma-ray peaks at 605 and 661 keV, respectively. The averages of the detection limits (3 standard deviations) of the ^134^Cs and ^137^Cs measurements were calculated to be 0.53 and 0.20 Bq m^−3^, respectively. In the case of the 605 keV photopeak from ^134^Cs, the cascade summing effect was corrected. The factor for the summing effect was about 2, which was calculated as the difference between the ^134^Cs/^137^Cs ratios at a distance of 15 cm from the detector and in the well hole of the detector. The averages of the analytical uncertainties (standard deviations) for the ^134^Cs and ^137^Cs measurements were calculated to be 13% and 7%, respectively. These uncertainties arose from the gamma counting, the calibration, and the correction for the summing effect. The radioactivity of ^137^Cs in a certified reference material for radionuclides, a water sample from Irish Sea (IAEA-443)^52^, was measured in the JAMSTEC laboratory. Results (0.36 ± 0.02 Bq kg^−1^, decay-corrected to 1 January 2007) agreed well with the radioactivity of ^137^Cs in the certified seawater. The radiocesium activity was also measured in the LLRL/KU laboratory with low-background Ge-detectors^51^,^53^. The averages of the detection limits for the ^134^Cs and ^137^Cs measurements in the LLRL/KU laboratory were 0.16 and 0.05 Bq m^−3^, respectively. The averages of the analytical uncertainties for ^134^Cs and ^137^Cs were calculated to be 11 and 6%, respectively. In the NIRS laboratory, the radiocesium activity was measured with Ge-detectors (GX-2019, Canberra Industries, Inc.). The uncertainties of radiocesium measurements in the NIRS laboratory (14% and 6% for ^134^Cs and ^137^Cs, respectively) were nearly equal to those in the JAMSTEC and LLRL/KU laboratories. The detection limits (2.2 Bq m^−3^ and 1.4 Bq m^−3 ^for ^134^Cs and ^137^Cs, respectively), however, were higher than those in the JAMSTEC and LLRL/KU laboratories. Measurements of ^134^Cs and ^137^Cs activities in AMP/Cs compounds derived from certified reference materials (IAEA-443 and 445), which were prepared by KANSO, among the three laboratories resulted in good agreement within uncertainties. This agreement confirmed the comparability of the radiocesium measurements at the three laboratories.

## Author Contributions

Y.K. and S.K. wrote the paper. T.K. supervised the project. Y.K. and A.M. designed the study and led the cruise. Y.K., M.A., Y.H. and T.A. performed the radiocesium measurements.

## Supplementary Material

Supplementary Information Table S1
